# Significant Factors in Cranial Remolding Orthotic Treatment of Asymmetrical Brachycephaly

**DOI:** 10.3390/jcm9041027

**Published:** 2020-04-05

**Authors:** Tiffany Graham, Kelly Millay, Jijia Wang, Beverley Adams-Huet, Elizabeth O’Briant, Madison Oldham, Shacoya Smith

**Affiliations:** 1 Health Care Sciences, Prosthetics-Orthotics Program, University of Texas Southwestern Medical Center, 6011 Harry Hines Blvd, Dallas, TX 75390-9091, USA; 2 Applied Clinical Research, University of Texas Southwestern Medical Center; 6011 Harry Hines Blvd, Dallas, TX 75390-9091, USA

**Keywords:** plagiocephaly, brachycephaly, asymmetrical brachycephaly, cranial flattening, cranial vault asymmetry index (CVAI), cephalic index (CI), cephalic ratio (CR), cranial orthosis, cranial helmet

## Abstract

This retrospective chart review focuses on determining the most effective time to begin cranial remolding orthosis (CRO) treatment for infants with asymmetrical brachycephaly. Subjects with asymmetrical brachycephaly started CRO treatment between 3 and 18 months of age. These infants had a cranial vault asymmetry index (CVAI) ≥ 3.5 and a cranial index (CI) ≥ 90. Subjects were excluded if they had any comorbidities affecting growth, dropped out of treatment, were lost to follow-up, or were noncompliant. Factors which were found to statistically influence treatment outcomes were subject initial age, initial CVAI, and initial CI. Overall, younger subjects were more likely to achieve a corrected head shape. The presence of prematurity or torticollis had statistically nonsignificant effects on the success of treatment. Initial CI was found to be a stronger predictor than initial CVAI as to which subjects achieved correction. The less severe the starting CI, the more likely the subject was to achieve full correction. The clinical understanding is that it requires more cranial growth to “round out” a full posterior skull flattening than an asymmetry. Based on the study results, infants with asymmetrical brachycephaly should be treated as early as possible to increase chances of achieving full correction of the deformity.

## 1. Introduction

Skull deformations reportedly affect around 45% of newborn babies [1] and can either be acquired congenitally or during infancy. Many skull deformations, such as plagiocephaly and brachycephaly, are acquired after the baby is born and are classified as positional skull deformities. For this study, asymmetrical brachycephaly is defined as a combination of the cranial shapes described by plagiocephaly and brachycephaly. “Plagiocephaly, or “oblique head”, is a deformity characterized by the flattening of one side of the posterior section of the head with compensatory ipsilateral frontal bossing, meaning that flattening of the occipital portion of the skull is seen with compensatory bossing ipsilaterally” [1]. “[Brachycephaly] is characterized by flattening of the entire occipital part of the skull, resulting in the shortening of the anterior-posterior dimension and the compensatory expansion of the medial-lateral dimension” [1]. Therefore, asymmetrical brachycephaly is characterized by a posterior flattening of the occiput as well as an asymmetry with compensatory posterior bossing contralaterally. These deformities are caused by external pressures on the baby’s skull when the skull is in the same position for extended periods of time [1,2,3,4,5]. This relatively common occurrence is often attributed to the American Academy of Pediatrics “Back to Sleep” campaign, which recommended infants be placed supine to sleep.

While repositioning therapy can be an effective treatment for some children, cranial remolding orthoses (CROs) are commonly used to help address all kinds of skull deformations, including asymmetrical brachycephaly [6]. Repositioning therapy usually involves keeping an infant’s head in a desired position. Parents are also advised to increase supervised tummy time and decrease time in car seats and rockers [7]. Depending on the type of deformational head shape and presence of torticollis (a congenital muscular dystrophy involving shortening of the sternocleidomastoid often associated with plagiocephaly [1]), repositioning can be difficult. If repositioning therapy is attempted and is not successful, or if the skull deformity is very severe to begin with, a CRO is then considered [6]. Severity has also been shown to play a role in the outcome of the final head shape in brachycephalic and plagiocephalic head shapes [3,8].

The validity of using CROs on infants with asymmetrical brachycephaly is still debated due to a lack of research in this area [4,5,9,10]. The goal of a CRO is to redirect the growing skull into the areas where the head is flattened [11,12,13,14]. During the first year of life, a baby’s skull is growing very quickly. Early intervention is thought to be an important step to having a more successful treatment of the skull deformation. The average age of babies starting CRO treatment is 7.6 months, while the average time spent in an orthosis is 3.7 months [1], but some studies have shown treatment prior to six months of age is preferable [2,12,15]. Treatment is normally discontinued when the family is happy with their child’s appearance or the child’s head has stopped growing at a fast enough rate to make substantial progress [16]. Generally, the upper limit of age is between 12 and 18 months with 18 months being the absolute limit as regulated by the Food and Drug Administration (FDA) in the United States [17].

The treatment of skull deformations is necessary to avoid certain medical and psychosocial issues in the child’s life. Abnormal head shapes in general can create difficulty for individuals wearing certain protective headwear, like bike helmets, and for ability to wear glasses [1]. Some studies have shown relationships between children with plagiocephaly and brachycephaly having additional health issues, with asymmetrical brachycephaly having the highest risk of complications [2]. Some of the issues associated with skull deformations include: increased need for special services at school age [1], delays in psychomotor development [18], abnormal speech development [1], jaw asymmetry [3], temporomandibular joint dysfunction [3,4,19,20,21,22,23], abnormal muscle tone [3], ear misalignment [24], and middle ear abnormalities [24]. Additionally, abnormal head and face shapes can be very noticeable on children and adults. The psychosocial consequence of having an abnormal face or head shape could result in bullying and low self-esteem. Research suggests that preventing and altering skull deformations would be beneficial to a child’s overall physical health and the psychosocial aspect of their lives [15,25,26].

In the literature, there are many existing scales for defining plagiocephaly and brachycephaly respectively, but none are for asymmetrical brachycephaly specifically. Asymmetrical brachycephaly is often grouped with plagiocephaly in the literature and thus has the same severity scales applied to it; however, using a plagiocephaly severity scale does not take into account the severity of the abnormal width to length proportion of the skull. There is a lack of studies on the asymmetrical brachycephaly population in particular as most of the studies examining asymmetrical brachycephaly are investigating deformational head shapes as a whole [10].

A commonly used measurement of the broadness of the head proportion is the Cranial Index (CI), which is also referred to as the Cranial Proportional Index or Cephalic Index. The CI is the measured width of the head divided by the length of the head multiplied by 100 and reported as a percentage. Normal ranges are usually reported between 75% and 85% [8,11]. Brachycephaly is when the CI is higher than the normal range, but “normal” also has a large range in the literature. Brachycephaly has been defined as a CI ≥ 80% [27], CI ≥ 82% [28], CI ≥ 93% [29], 95% < CI < 104% [11], and CI ≥ 97% [16]. Some clinicians use two standard deviations from the published mean to define brachycephaly [30]. One study categorized CI as mild, moderate, and severe based on the percentiles for their ages with mild being above the 75th percentile, moderate above the 90th percentile, and severe above the 97th percentile [31]. One issue with this measurement scale is that supine sleep position has shifted the average head shape closer to brachycephalic in recent years [9]. This has changed what society views as a normal head shape.

**Figure 1:**
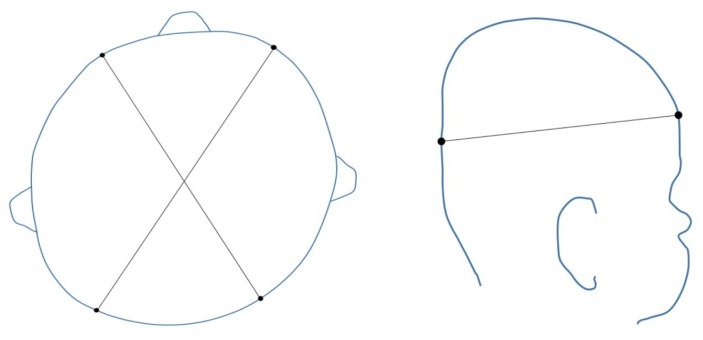
Illustration of cranial measurement locations. The superior view on the left illustrates the diagonal measurement locations used in the calculation of the cranial vault asymmetry index (CVAI). The absolute value of the difference of these two diagonal measurements is defined as the cranial vault asymmetry (CVA). The lateral view on the right illustrates the location of the greater equator of the skull, where diagonal measurements are taken.

The severity of plagiocephaly can be categorized by the number of quadrants involved in the skull deformation and if there is a shift in the ear or eye placement [24]. Commonly used measurements of asymmetry are the Cranial Vault Asymmetry (CVA) and Cranial Vault Asymmetry Index (CVAI). The CVA is the absolute value of the difference of the cranial diagonals and CVAI is this CVA divided by the longer diagonal multiplied by 100. Therefore, the CVAI is a measurement of the CVA in relationship to the overall size of the head. Measurements are taken at the greater equator of the skull, as shown in Figure 1.

When using CVA, a difference of up to 3mm is usually considered within normal limits, a difference between 3mm and 12mm is mild to moderate, and severe is any difference greater than 12mm; however, these scales have not been validated in the literature [2,32]. For this study, CVAI was chosen for cranial measurement reporting due to the availability of the validated Children’s Healthcare of Atlanta (CHOA) scale [7]. The CHOA scale defines plagiocephaly as mild when CVAI is 3.5–6.25, moderate when CVAI is 6.25–8.75, severe as a CVAI 8.75–11, and very severe as greater than 11 [7]. Wilbrand et al. (2017) published a table of normative values of circumference, width, length, CVA, CI, and CVAI for infants from 0 to 24 months which can be used to help develop standards for defining plagiocephaly and brachycephaly [10]. This in turn can help develop standards for defining asymmetrical brachycephaly [16]. Both CI and CVAI are useful for what they were designed to measure: brachycephaly and plagiocephaly, respectively. However, there is no universal scale to measure asymmetrical brachycephaly which essentially functions as a combination of both of these deformities. Some clinicians use a combination of these scales, i.e., if a patient has a moderate CVAI and a mild CI they have a moderate deformity [2].

Many studies have examined the effectiveness of CROs, however, no definite conclusions have been reached. Although the most beneficial time to start and stop treatment for all skull deformations is still debated in the literature, the evidence seems to suggest that treatment should begin at the youngest age possible for maximal success [24,32,33]. Only a small fraction of the limited research about skull deformations available today pertains to asymmetrical brachycephaly. The trend of greater correction with treatment of deformational head shapes (plagiocephaly and brachycephaly) with younger starting age is likely true with asymmetrical brachycephaly. However, this has not been fully examined in the literature. Therefore, the treatment of this condition relies mostly on clinical experience and varies greatly. For that reason, a retrospective chart review was performed to assess the outcomes of infants diagnosed with asymmetrical brachycephaly treated with CROs at various starting ages and severities.

This study will aim to determine the effectiveness of CRO treatment in terms of treatment duration and final head shape measurements in relationship to starting age and cranial measurements in infants with asymmetrical brachycephaly with a goal of answering the clinical question: what is the most effective starting age for CRO treatment for asymmetrical brachycephaly? Specifically, the authors sought to determine which factors (prematurity, torticollis, initial starting age of treatment, initial severity of CI, and initial severity of CVAI) play a significant role in correction of the deformity. There is an additional research question of whether the severity of the brachycephalic portion (measured by CI) or plagiocephalic portion (measured by CVAI) of the asymmetrical brachycephalic head shape has more influence on correction as this is of clinical interest.

## 2. Materials and Methods

This retrospective chart review was conducted in accordance with the Declaration of Helsinki and the protocol was approved with a waiver of consent by the University of Texas Southwestern Medical Center’s Institutional Review Board (IRB Number: STU 022017-046). A data transfer agreement was granted through Level 4 Prosthetics & Orthotics and University of Texas Southwestern Medical Center (Contract Number: DUA201806-0022). The charts reviewed were infants who underwent CRO treatment in one of the three Texas offices of Level 4 Prosthetics & Orthotics (one office in Addison, TX, USA and two offices in San Antonio, TX, USA).

In this retrospective chart review, information from 500 subjects was gathered from patient charts via electronic document access performed at Level 4 Prosthetics and Orthotics (now RestorePOC) in Addison, TX, USA. All three offices of Level 4 Prosthetics and Orthotics trained their clinicians in the same methods of treating asymmetrical brachycephaly and utilized the same method of fabricating CROs. All clinics used a STARscanner (Orthomerica Orlando, FL, USA) for initial and final scans of the head shape. Measurements were taken using the STARscan report (Cranial Comparison Utility software, Vorum Research Corporation, Vancouver, BC, Canada) at Level 3 which generally is the greater equator of the skull. Additionally, all subjects used the Orthomerica STARband (Orthomerica Orlando, FL, USA) as the brand of CRO.

In order to be included in the study, subjects had to meet the following criteria: the subjects needed to be diagnosed with a deformational head shape by a pediatrician, craniofacial surgeon, neurosurgeon, or plastic surgeon and be treated at one of the three Level 4 Prosthetic and Orthotic clinics covered by the IRB. In order to fit this study’s definition of asymmetrical brachycephaly, the subjects had to have a CVAI of 3.5 or greater and a CI of 90% or greater. Treatment had to have been started and completed between January 2013 and June 2017. Subjects had to start treatment between 3 and 18 months of age as regulated by the FDA. Subjects born prematurely were included in this study and their age was adjusted to reflect their developmental age. Subjects were considered premature if they were born at 37 weeks gestation or earlier. Treatment initiation age was calculated as the nearest half month postpartum, corrected for prematurity. Corrected age was calculated by taking the number of weeks of prematurity and subtracting this from the postpartum age, then rounding to the nearest half month. Subjects with torticollis were also included in this study as the incidence of torticollis is highly correlated with asymmetrical brachycephaly. The patients with torticollis were instructed by the orthotist treating them to receive treatment for their torticollis concurrently as part of their cranial remolding treatment of asymmetrical brachycephaly.

Subjects were excluded if they had any synostotic head shape or positional deformity other than asymmetrical brachycephaly; had any comorbidities that would affect their growth or head shape such as Down Syndrome, heart defects, or feeding problems; dropped out of treatment; were lost to follow up; completed treatment at a facility not covered by the IRB, had incomplete data, or were noncompliant. Noncompliance was based on either parent reported noncompliance or clinician documented suspicions of noncompliance.

**Figure 2:**
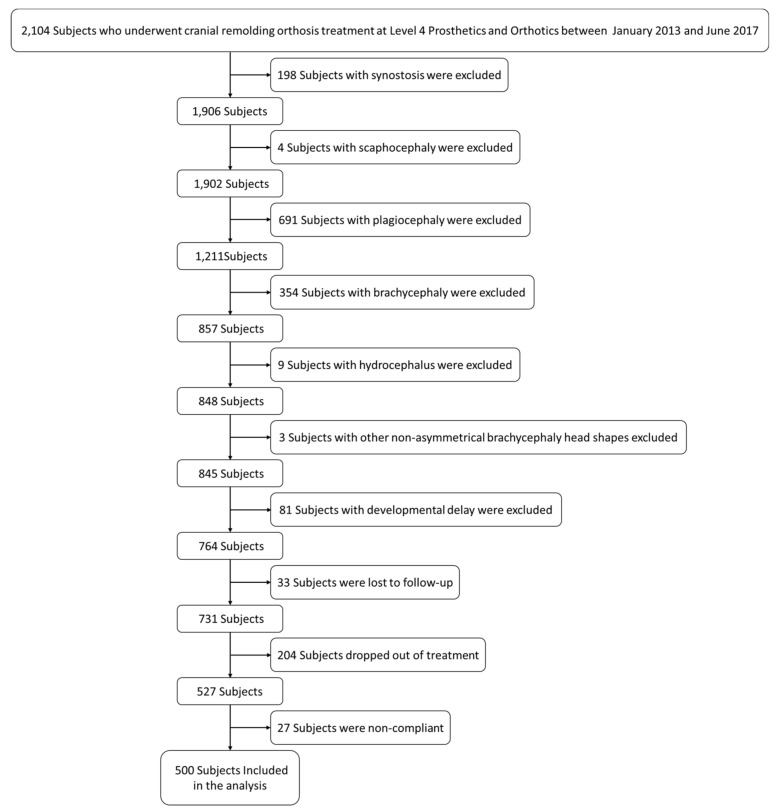
Flowchart showing the reduction from 2104 subjects to the 500 subjects included in the analysis based on the study inclusion and exclusion criteria.

In total, 2104 charts were reviewed, and 500 subjects were included. Figure 2 shows the process of exclusion. Of the 1604 subjects whose charts were excluded, 198 had synostosis, four had scaphocephaly, 691 had plagiocephaly, 354 had brachycephaly, nine had hydrocephalus, three had nonasymmetrical brachycephaly head shapes, 81 had comorbidities or developmental delay, 33 were lost to follow up, 204 dropped out of treatment, and 27 were noncompliant.

The protocol of the study was that all charts from January 2013 to June 2017 from Level 4 Prosthetics and Orthotics were reviewed to find 500 subjects who met the inclusion criteria. Subjects all consented to treatment at Level 4 Prosthetics and Orthotics at the time of their evaluations and a waiver of consent was granted by the IRB for this retrospective chart review because no identifiable patient data was included in this study. The patient chart information gathered was study identification numbers, corrected age at start and end of treatment, presence or absence of prematurity, starting and ending CVAI, CVAI difference, starting and ending CI, CI difference, presence or absence of torticollis, developmental comorbidities, and treatment time. All data were stored on encrypted flash drives. The decryption key, which could convert patient identification numbers used by Level 4 Prosthetics and Orthotics to the study identification numbers, was kept in a secure location at Level 4 Prosthetics and Orthotics. At the conclusion of the study, the decryption key was destroyed and no identifiers remained.

Data was collected and initial calculations were completed using Microsoft Excel (Microsoft Corporation, Redmond, WA, USA). Data analysis was conducted using SPSS (IBM Analytics Armock, New York, NY, USA) and SAS (SAS Inc., Cary, NC, USA). The goal of the data analysis was to find the group of patients who are most likely to achieve full correction (i.e., which severity or age group) and to find the most effective start age(s) for treatment. The dependent variable was correction of the head shape which was measured by the CVAI and CI at the end of treatment. The independent variables were the subjects’ corrected age, whether or not they had torticollis, whether or not they were premature, and the subjects’ initial severity measured by starting CVAI and CI. There are possible confounding variables with the most likely being how compliant the subjects were with their 23 h per day wear schedule or possible undiagnosed developmental comorbidities affecting growth. These were controlled for (to the best of our ability) by excluding subjects who were documented by clinicians in their charts to be noncompliant in wearing the CRO and excluding subjects who were suspected to have a developmental or growth issue by their clinician as written in the patient chart, respectively.

Subjects were grouped according to severity. Although the CHOA scale [7] is used to describe plagiocephalic head shapes, no uniform scale is accepted for brachycephalic head shapes, and no scale exists for a combination of the two deformations, as seen in asymmetrical brachycephaly. Therefore, for this study, a brachycephalic scale was created based on a variety of published scales in the literature [11,27,28,29,30,31], combined with the authors’ clinical expertise. This scale defines CI < 90% as normal, 90% ≤ CI ≤ 93% as mild, 93% < CI ≤ 97% as moderate, and CI > 97% as severe. A matrix was created using the CHOA scale on the *y*-axis to describe the severity of the CVAI and the described CI scale on the *x*-axis to describe the severity of the CI.

Descriptive statistics were employed for summarizing variables, which can be found in the Supplementary Table S1. Logistic regressions and receiver operating characteristic (ROC) curves were performed to investigate the variables that significantly affected head shape correction. Odds ratios were also presented to evaluate the association between different variables and correction. The simple linear regression was used to calculate the average measurement change per month. The level of significance was set at 5%.

## 3. Results

Table 1 shows the number of subjects within 20 categories of severity based on cranial measurements at the start of treatment. This is a way to visualize where the subjects fell on the combination of both scales (CI severity for posterior flattening and CVAI for asymmetry). The demographic distribution is not evenly balanced; there are fewer patients in the more severe categories. Based on the inclusion criteria, no infants began treatment in the normal range. Table 2 shows the number of subjects within the same 20 categories based on severity at the end of treatment. This showed that there was a general trend of the infant’s head shapes shifting toward normal or mild on both scales. Additionally, the number of infants in the Severe CI or Very Severe CVAI categories drastically reduced, showing that these subjects improved.

Table 3 includes individuals that achieved full correction on the CHOA and CI scale. When determining which subjects achieved full correction, any subject with a CI less than or equal to 90% and a CVAI less than or equal to 6.25 was considered to have achieved full cranial correction as this benchmark was similar to the training clinicians received (clinical goal was to get the CVA below 6mm and CI below 90%). Overall, 44.60% of patients achieved full correction and a majority of patients (85.60%) ended with scores of normal or mild on the CHOA and CI scales, which supports the efficacy of CRO treatment. Additionally, this shows that subjects with a severe CI at the start of treatment did not usually achieve full correction. By comparing Table 1 to Table 3, it can be seen that only 2 of the 107 subjects in the Severe starting CI category achieved full correction, compared to 67 of the 205 subjects in the Moderate starting CI category and 154 of the 188 subjects in the Mild starting CI category.

**Figure 3:**
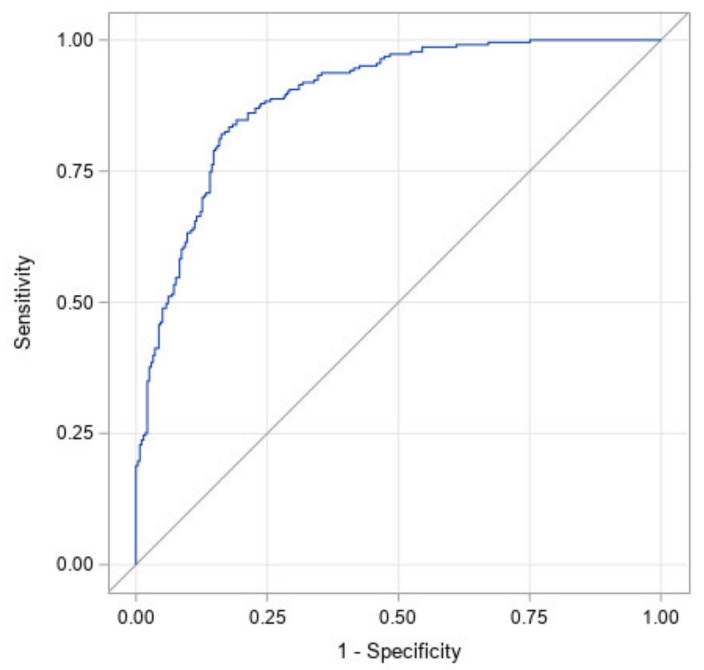
Receiver operating characteristic (ROC) curve for a model with all factors. Factors are: initial cephalic index (CI), initial cranial vault asymmetry index (CVAI), interaction between initial CI and CVAI, initial age, prematurity, and torticollis). The area under the curve is 0.89.

Figure 3 illustrates the ROC curve for a model with all factors: prematurity, torticollis, corrected age at start of treatment, CVAI at start of treatment, CI at start of treatment, and the interaction between CI and CVAI at the start of treatment. It has an area under the curve of 0.89 with a 95% confidence interval of (0.87, 0.92). This high area under the curve illustrates that this study likely identified the most significant factors in the success of CRO treatment for asymmetrical brachycephaly. Table 4 shows the significance of each factor in the ROC curve. The effects of initial age, starting CI, as well as the interaction between the starting CI and starting CVAI were significant at *p* <0.05. The effects of prematurity and torticollis are not statistically significant. Since prematurity was not significant, this indicates that the correction made for prematurity in this study was an accurate representation of the premature subjects’ growth. The most significant factor is CI at start followed by corrected age at start. This can be seen through their exact *p*-values and indicates that starting CI is the most important factor in predicting correction.

**Figure 4:**
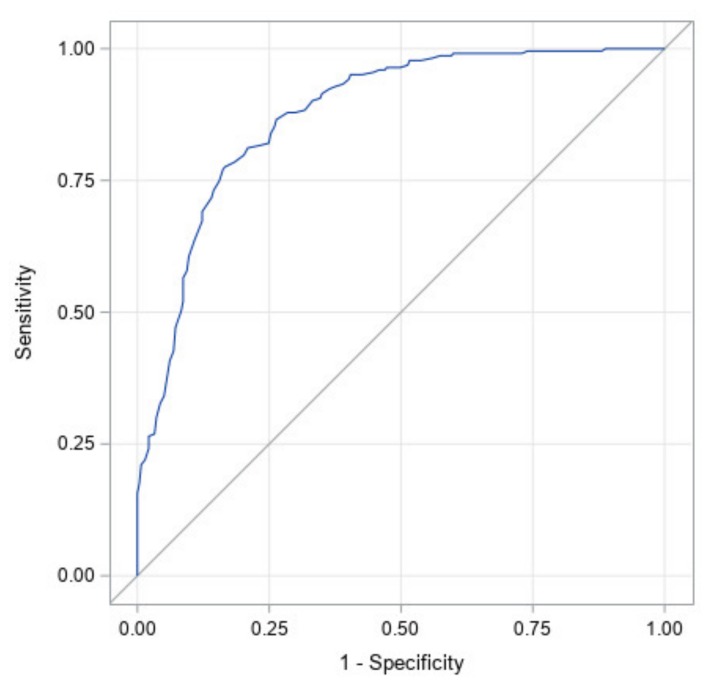
ROC curve for the initial cephalic index only. The area under the curve is 0.88 (statistically significant with *p* <0.0001).

Figure 4 illustrates the ROC curve for CI at start of treatment only. It has an area under the curve of 0.88 with a 95% confidence interval of (0.85, 0.91). The high area under the curve and narrow confidence interval indicates that starting CI was found to be the largest determinant of which infants would achieve full correction.

**Figure 5:**
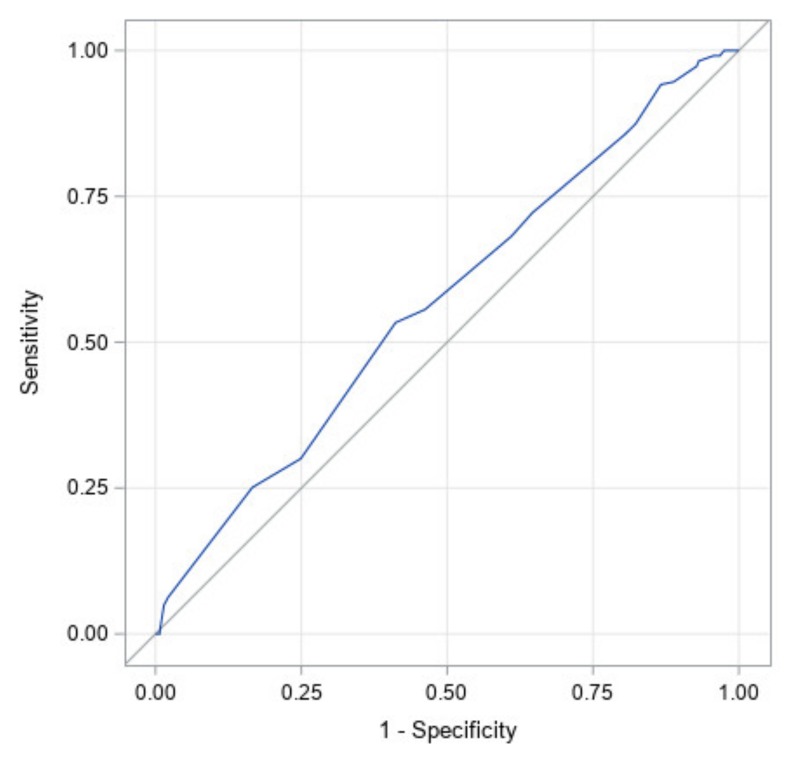
ROC curve for initial age only. The area under the curve is 0.57 (statistically significant with *p* = 0.002).

Figure 5 illustrates the ROC curve for the corrected age at the start of treatment. It has an area under the curve of 0.57 with a 95% confidence interval of (0.52, 0.62). This is the second largest area under the curve for an individual factor and therefore was found to be the second largest predictor of which subjects would achieve full correction to the head shape.

**Figure 6:**
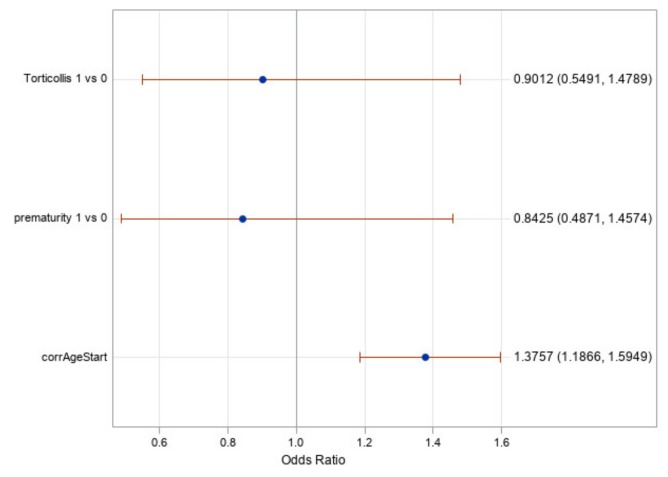
Odds ratios with 95% confidence intervals for torticollis, prematurity, and corrected age at start of treatment.

Figure 6 shows the odds ratios for torticollis, prematurity, and the starting age (corrected for prematurity). It shows once again that torticollis and prematurity are not significant factors as their odds ratio confidence intervals include one. It does show, however, that the corrected age of initiation of treatment is a significant factor with an odds ratio value of 1.38. This means that for every month earlier a subject began treatment, the odds of achieving a full cranial correction is approximately 1.38 times greater. It is worth noting that age in this statistical test was treated as a continuous variable, which gives greater credence to the results found.

Table 5 shows the linear regression estimates for the change of CI and CVAI per month. This provides further evidence that subjects improved even if they did not reach full correction. However, these results should be put into the context of the overall study. Based on Table 5, if a subject started at a CI of 91.1% and a CVAI of 6.6 and the clinical goal was a CI of less than 90% and CVAI of less than 6.25, then this subject would have been in treatment for approximately six months (91.1% − 6 × 0.2% = 89.9% and 6.6 – 6 × 0.07 = 6.18). However, this estimate is based on the average change over the entire course of treatment for all age groups included in this study. It is important to note that most patients corrected at much quicker rates, which is reflective of the previously listed results showing that the corrected age at the start of treatment significantly affected the treatment results. In general, younger infants had much quicker correction rates per month of treatment than older infants.

## 4. Discussion

Results from this study support the hypothesis that the effectiveness of CROs is related to both age at the initiation of treatment and severity of CI and CVAI at the start of treatment in asymmetrical brachycephaly. Age has been shown in previous studies of isolated plagiocephaly [32,33,34,35] and brachycephaly to significantly affect treatment [3,5]. This is intuitive as younger infants have more growth potential left which the CRO can harness to provide correction. According to Meyer-Marcotty et al.’s study in 2018 of infants in the 4–10 month age range, infants between 4 and 6 months have the greatest neurocranial volume increase in comparison to the older subjects [36]. The cranial circumferential growth chart published by the CDC shows younger infants have faster growth [37]. Additionally, previous literature has supported the conclusion that a younger age at initiation of treatment correlates to higher likelihood of correction for plagiocephaly and brachycephaly respectively [3,5,24,32,33,38,39]. The initial severity of the head shape plays a crucial role in determining which subjects achieved full correction and how much correction they achieved. More severe head shapes are less likely to fully correct as has been demonstrated in the literature for isolated plagiocephaly and isolated brachycephaly [1,3,8]. It is important to note that in this study, all patients did improve with CRO treatment.

In this study, severity was graded in a matrix of CVAI and CI severities as shown in Table 1 and Table 2. One of the questions of this study was whether the severity of the brachycephalic portion (measured by CI) or plagiocephalic portion (measured by CVAI) of the asymmetrical brachycephalic head shape had more influence on correction. The results show both factors were statistically significant and had an effect on correction. However, the initial CVAI had a higher *p*-value than initial CI, indicating initial CVAI is not as influential as initial CI in determining which infants achieved full cranial correction—a result which is further reflected in Table 3. Therefore, the initial CI is the most significant factor in the model to predict whether or not the subjects will achieve full correction. These clinically significant findings demonstrate that in our retrospective chart review, the initial CI was the single most important factor in determining if a subject achieved full correction. When discussing this result with clinicians, this was determined to be reasonable as it requires more cranial growth to “round out” a full posterior flattening of the skull (i.e., a brachycephalic deformation) than a unilateral posterior-lateral flattening of the skull (i.e., a plagiocephalic deformation) [5]. Clinically, this is important as it shows that an increased CI is more difficult to correct and therefore should be treated sooner. It also raises questions about how treatment protocols for asymmetrical brachycephaly should be structured to give the greatest chance of achieving full correction.

It is worth remembering that the large area under the ROC curve for CI could be a self-fulfilling prophecy. The inclusion criteria of this study required subjects to have a CI greater than or equal to 90% and a CVAI greater than or equal to 3.5 to be considered asymmetrical brachycephalic but required the CVAI to only be 6.25 or below and CI of 90% or below to be a fully corrected head shape. This meant that many subjects already had a “fully corrected” CVAI value from the start, even if their CVAI value did not change significantly. Therefore, it is inherently likely the CI value will drive whether or not the subjects achieved full correction based on the model. In response to this finding, Table 5 shows the typical CI and CVAI change per month during treatment. The reasoning behind the difference in inclusion and “fully corrected” criteria was that clinicians in these three offices were taught to get the CVA below 6mm to be considered within normal limits despite CVAI < 6.25 being considered mild plagiocephaly according to the CHOA scale. At this point, the clinicians often would discontinue treatment. Therefore, if our criteria for full correction followed the CHOA scale at less than 3.5 CVAI, almost no subjects would achieve this correction as this was not the clinical goal and hence patients were often discharged from treatment prior to reaching this measurement. Future prospective studies should define more distinct inclusion and correction criteria with clinicians understanding treatment goals as the correction criteria in order to avoid this bias.

All offices included in the study were trained to use the same method to treat asymmetrical brachycephaly. Their method involved generally correcting the asymmetry (reducing the CVAI) first and then rounding out the flatness in the back (reducing the CI). However, the clinicians were taught to treat whichever part of the head shape was “worse” which means that not every patient was treated with exactly the same protocol. The CVAI and CI were not generally treated concurrently due to either the expectation of limited growth (a concern with older patients) or because of the limitations of needing places for the CRO to “hold” or be tight on the subject’s head. According to clinicians, if too many reliefs were made for the subject’s head to grow into and correct, the orthosis had a strong potential to rotate, causing issues with the fit and presumably lessening the effectiveness of the orthosis. This method of (generally) addressing the CI after the CVAI has been corrected would mean that if the patient ran out of growth potential or the parents wanted to discontinue treatment, full correction may not be seen in the CI. This is a possible confounding variable in why the initial CI was so highly correlated with full cranial correction.

The analysis of effects and odds ratio results in Table 4 and Figure 6 showed that the effect of prematurity is nonsignificant; therefore, we can conclude that this modeling of subtracting the weeks of prematurity from the postpartum age of the subject appears to be accurate and should be considered clinically when predicting the treatment time or likelihood of successful treatment. Additionally, prematurity is not a contraindication to CRO treatment as age-adjusted premature infants corrected similarly to their peers born at full term.

In the same vein, the effect of the presence of torticollis was found to be nonsignificant in our analyses (reference Table 4 and Figure 6). Therefore, despite torticollis being a risk factor in developing a deformational head shape [40,41], it does not appear to affect the likelihood or ability of infants to achieve full cranial correction as long as it is concurrently treated. This result is similar to what the authors have previously shown in treatment of isolated plagiocephaly [35]. All subjects in this study were being treated for torticollis during their CRO treatment if they had been diagnosed with torticollis. All of this should be interpreted to mean that a diagnosis of torticollis at the beginning of CRO treatment for asymmetrical brachycephaly has a nonsignificant effect on the success of a patient, provided that the patient is undergoing concurrent CRO treatment and torticollis treatment.

Limitations of this study include patient compliance, different CVAI values for inclusion and full cranial correction, no established validated brachycephaly or asymmetrical brachycephaly scale, differing treatment protocols, possible comorbidities, a single CRO brand, uneven distribution of ages and severities, the variability of individual growth rates, the examination of only the isolated asymmetrical brachycephalic head shape, and the use of a single scanner type. As this was a retrospective chart review, no control group was able to be studied. The generalizability of the results of this study to other CRO brands and treatment protocols should be determined by future studies. This study could be enhanced by increasing the number of included subjects, particularly subjects with more severe initial measurement severities.

## 5. Conclusions

In this study, we concluded that younger age at initiation of treatment correlates to a higher likelihood of full correction for asymmetrical brachycephaly. This corresponds to the previous findings in the literature for plagiocephaly and brachycephaly respectively [3,5,24,32,33,38,39]. Overall, this illustrates that in terms of clinical significance, infants with asymmetrical brachycephaly who are indicated for CRO therapy should be treated with a CRO as early as possible, once they can support their neck (and after they turn three months of age, in accordance with FDA regulations) to give them the greatest chance of achieving full cranial correction. Additionally, the initial severity of the CI was a strong predictor of the efficacy of CRO treatment in achieving full cranial correction. Prematurity, as long as it was age-corrected, and torticollis, as long as it was concurrently treated, were nonsignificant factors in achieving full correction. Clinically, these patients are candidates for CRO therapy and should be expected to correct similarly to their unaffected peers.

## Figures and Tables

**Table 1:** Number of infants within each cranial severity category at the start of treatment based on the initial cranial vault asymmetry index (CVAI) and cephalic index (CI).

		Cranial Index (CI) Scale Severity				
		Normal CI < 90%	Mild 90% ≤ CI ≤ 93%	Moderate 93% < CI ≤ 97%	Severe CI > 97%	Total
**Children’s Healthcare of Atlanta (CHOA) Scale Severity**	Normal CVAI < 3.5	N/A	N/A	N/A	N/A	N/A
	Mild 3.5 ≤ CVAI < 6.25	N/A	59	97	54	210
	Moderate 6.25 ≤ CVAI < 8.75	N/A	86	78	40	204
	Severe 8.75 ≤ CVAI < 11	N/A	34	23	10	67
	Very Severe CVAI ≥ 11	N/A	9	7	3	19
	Total	N/A	188	205	107	500

**Table 2:** Number of infants within each cranial severity category at the end of treatment based on the initial cranial vault asymmetry index (CVAI) and cephalic index (CI).

		Cranial Index (CI) Scale Severity				
		Normal CI < 90%	Mild 90% ≤ CI ≤ 93%	Moderate 93% < CI ≤ 97%	Severe CI > 97%	Total
**Children’s Healthcare of Atlanta (CHOA) Scale Severity**	Normal CVAI < 3.5	108	114	28	4	254
	Mild 3.5 ≤ CVAI < 6.25	104	102	27	3	236
	Moderate 6.25 ≤ CVAI < 8.75	5	2	2	0	9
	Severe 8.75 ≤ CVAI < 11	0	1	0	0	1
	Very Severe CVAI ≥ 11	0	0	0	0	0
	Total	217	219	57	7	500

**Table 3:** Number of subjects who achieved full cranial correction in each starting severity category ^1^, based on the initial cranial vault asymmetry index (CVAI) and cephalic index (CI). Full correction was defined as a CVAI ≤ 6.5 and CI ≤ 90%.

		Cranial Index (CI) Scale Severity			
		Mild 90% ≤ CI ≤ 93%	Moderate 93% < CI ≤ 97%	Severe CI > 97%	Total
**Children’s Healthcare of Atlanta (CHOA) Scale Severity**	Mild 3.5 ≤ CVAI < 6.25	52 (88.14%)	28 (28.87%)	0 (0%)	80 (38.10%)
	Moderate 6.25 ≤ CVAI < 8.75	70 (81.40%)	26 (33.33%)	2 (5.00%)	98 (48.04%)
	Severe 8.75 ≤ CVAI < 11	25 (73.53%)	10 (43.48%)	0 (0%)	35 (52.24%)
	Very Severe CVAI ≥ 11	7 (77.78%)	3 (42.86%)	0 (0%)	10 (52.63%)
	Total	154 (81.91%)	67 (32.68%)	2 (1.87%)	223 (44.60%)

^1^ Number and percentages refer to the percent of subjects in that category at start of treatment who achieved full cranial correction (i.e., corresponding values from Table 1).

**Table 4:** Analysis of effects for Figure 3.

Effect	*p*-Value
Prematurity	0.54
Torticollis	0.68
Initial Age	<0.0001 *
Starting Cranial Vault Asymmetry Index (CVAI)	0.02 *
Starting Cephalic Index (CI)	<0.0001 *
Interaction between Starting CVAI and Starting CI	0.02 *

* Indicates significance at *p* ≤0.05.

**Table 5:** Change per month estimates for CI and CVAI across 500 subjects of varying ages.

	Change per Month Estimate
**Cranial Index (CI)**	−0.20% per month
**Cranial Vault Asymmetry Index (CVAI)**	−0.07 per month

## Supplementary Materials

The following are available online at https://www.mdpi.com/2077-0383/9/4/1027/s1, Table S1: Descriptive statistics of the 500 included subjects.
