# Supplementary material for: Significant Factors in Cranial Remolding Orthotic Treatment of Asymmetrical Brachycephaly
Source: J Clin Med. 2020 Apr 5;9(4):1027. doi: 10.3390/jcm9041027 (PMC7231243; doi:10.3390/jcm9041027)
Supplement: Supplementary file 1 [file jcm-09-01027-s001.pdf]

Descriptive Statistics						
	**Range**	**Minimum**	**Maximum**	**Mean**	**Std. Deviation**	**Variance**
**Corrected Age at Start (months)**	15.00	2.00	17.00	5.95	1.97	3.89
**Corrected Age at End (months)**	17.00	5.00	22.00	10.32	2.85	8.11
**Cranial Vault Asymmetry Index (CVAI) Start**	11.20	3.50	14.70	6.85	2.08	4.33
**Cranial Vault Asymmetry Index (CVAI) End**	10.60	0.10	10.70	2.90	1.40	1.95
**Cephalic Index (CI) Start (%)**	19.80	90.00	109.80	94.67	3.29	10.80
**Cephalic Index (CI) End (%)**	21.50	81.30	102.80	90.54	2.39	5.70
**Treatment Time (months)**	10.00	1.00	11.00	4.30	1.67	2.78
**Cranial Vault Asymmetry Index (CVAI) Difference**	13.40	0.20	13.60	3.94	1.79	3.20
**Cephalic Index (CI) Difference (%)**	12.90	0.20	13.10	4.13	2.08	4.33
